# Delignification of corncob via combined hydrodynamic cavitation and enzymatic pretreatment: process optimization by response surface methodology

**DOI:** 10.1186/s13068-018-1204-y

**Published:** 2018-07-24

**Authors:** Kiruthika Thangavelu, Ramesh Desikan, Oxana P. Taran, Sivakumar Uthandi

**Affiliations:** 1 Department of Bioenergy, Agricultural Engineering College and Research Institute, Tamil Nadu Agricultural University, Coimbatore, Tamil Nadu 641003 India; 2 Department of Chemistry, Boreskov Institute of Catalysis, Novosibirsk, 630090 Russia; 3 Biocatalysts Lab, Department of Agricultural Microbiology, Tamil Nadu Agricultural University, Coimbatore, Tamil Nadu 641003 India

**Keywords:** Corncob, Pretreatment, Delignification, Hydrodynamic cavitation, Laccase enzyme

## Abstract

**Background:**

Renewable liquid biofuel production will reduce crude oil import of India. To displace the huge quantity of fossil fuels used for energy production, this research was focused on utilization of unexploited low-cost agricultural residues for biofuel production. Corncobs are a byproduct of corn processing industry, and till now it is not utilized for biofuel production, eventhough it has high lignocellulosic concent. In this study, combined hydrodynamic cavitation and enzymatic (HCE) method was evaluated as a pretreatment method of corncob for biofuel production. The most significant process parameters namely (i) enzyme loading (3–10 U g^−1^), (ii) biomass loading (2.5–5.0%), and (iii) duration (5–60 min) were optimized and their effects on combined HCE pretreatment of corncob was studied through response surface methodology for lignin reduction, hemicellulose reduction and cellulose increase.

**Results:**

The highest lignin reduction (47.4%) was obtained in orifice plate 1 (OP_1_) under the optimized conditions namely biomass loading at 5%, enzyme loading at 6.5 U g^−1^ of biomass, and reaction duration of 60 min. The above tested independent variables had a significant effect on lignin reduction. The cavitational yield and energy consumption under the above-mentioned optimized conditions for OP_1_ was 3.56 × 10^−5^ g J^−1^ and 1.35 MJ kg^−1^, respectively.

**Conclusions:**

It is evident from the study that HCE is an effective technology and requires less energy (1.35 MJ kg^−1^) than other biomass pretreatment methods.

**Electronic supplementary material:**

The online version of this article (10.1186/s13068-018-1204-y) contains supplementary material, which is available to authorized users.

## Background

Maize (*Zea mays* L.) is an important cereal grown in India for food and nutritional security, with a total production and productivity of 24 million metric tons and 10.2 t ha^−1^, respectively. It is estimated that about 180 kg of cobs are produced per ton of shelled maize, leading to huge amounts of corncob accumulation, which are left unutilized. Corncobs are a rich source of lignocellulosic material containing primarily cellulose (36%), hemicelluloses (26%), and lignin (17%). Currently, the corncobs are used for production of paper pulp due to its cellulosic contents. However, it can be a better source for biofuel production due to its constituents. It should be noted that corncob is not a food or feed substrate. Production of biofuels and other value-added industrially important fine chemicals from lignocellulosic biomass will not only lower our dependence on fossil fuels and green gas emissions but also would favor the development of sustainable biorefineries. Hence, utilization of corncob is the best resource for deriving useful industrial chemicals and corncob-based biofuel as one of the viable and promising approaches for sustainable utilization of natural resources and ensuring energy security.

Conversion of lignocellulosic biomass (LCB) into bioalcohols (bioethanol or biobutanol) involves three stages, viz, biomass pretreatment, hydrolysis, and fermentation and among these biomass pretreatment is an inevitable bioprocesses and consumes up to 40% of total production cost [1]. Currently, physical or chemical or biological methods or their combinations are employed for biomass pretreatment. The most common biomass deconstruction methods includes physical (grinding/milling [2, 3]), chemical (acid/alkaline [4, 5], ionic liquids [6], subcritical and supercritical water, organosolv [7]), biological (white rot fungi [8] or laccase enzyme [9]), and combinations of these methods (hot water/autohydrolysis [10, 11], steam explosion [11], supercritical CO_2_ [12] or ammonia fiber explosion [13]). Generally, combined pretreatment methods are preferred due to its better performance/efficiency in delignification compared to individual methods [14]. The biomass pretreatment methods which are used currently are more complex and energy intensive, eventually requires more efforts during scaling up process.

Recently, hydrodynamic cavitation (HC) technology has been successfully employed in diversified fields namely biodiesel production, wastewater treatment, food processing and different bioprocessing operations. Combined HC pretreatment and other chemical catalysts have been used for production of biofuel from different lignocellulosic feedstocks [15–18]. During HC pretreatment, highly reactive radicals (OH^−^ and H^−^) are formed in the working fluid due to the cavitation effect, which can cause deconstruction of lignin structures. Though HC technology has been considered as a viable pretreatment method for processing diverse feedstocks, it is not optimal for targeting specific end products.

The methodology adopted in HC pretreatment studies creates cavitation by continuous circulation of the working fluid (water containing biocatalyst) by a pump through an orifice plate of the reactor. However, the raw biomass has to be kept within or outside of the HC zone [19]. But in the present investigation, both the biomass and biocatalyst were circulated continuously throughout the reactor to enhance the bioconversion efficiency. The advantages of this approach are less energy requirement and operation at ambient conditions.

Furthermore, biocatalysts such as enzyme use phenoxy radicals for the removal of recalcitrant fractions of lignocellulosic biomass. The best-characterized lignin depolymerizing enzymes are multi copper oxidases namely laccases (EC 1.10.3.2), which utilizes O_2_ instead of H_2_O_2_ to mediate substrate oxidation and also not prone to cofactor degradation. To the best of our knowledge, combination of HC and enzyme (HCE) was not attempted so far. In this context, the study highlights the significance of combination of HC with laccase for pretreatment of corncob biomass, optimization of process variables, and their interaction effects on higher recovery of lignin from corncob.

## Results and discussion

### Optimization of HCE method

Optimization of HCE pretreatment of delignification was performed using orifice plate 1 (OP_1_) and orifice plate 2 (OP_2_) at an optimized inlet pressure of 50 and 100 kPa, respectively. To determine the optimum values of variables for HCE pretreatment, response surface methodology (RSM) was employed with all the input variables set in the range, with output responses set at maximum levels. For process optimization, three factors were evaluated for three different responses such as maximum lignin reduction (%), hemicellulose reduction (%) and cellulose increase (%) using Box–Behnken design in RSM, which includes biomass loading (*A*), enzyme loading (*B*), and time (*C*). These variables were statistically optimized using 3^3^ factorial design.

The optimized conditions for HCE pretreatment with orifice plate 1 (HCE-OP_1_), are biomass loading at 5%, enzyme loading at 6.5 U g^−1^ of biomass, and a process time of 60 min. The result showed a reduction in lignin (47.4%) and hemicellulose content (3.2%) and an increase in cellulose content (25.3%). Furthermore, it was observed that the biomass loading and duration have resulted in the optimum response with the higher values. However, the enzyme loading resulted in the mid value. The quadratic model of percentage of lignin reduction had a predicted and adjusted *R*^*2*^ values of 0.99 and 0.98, respectively. The predicted *R*^*2*^ value was in agreement with the adjusted *R*^*2*^ value, since the difference between two values was less than 0.02, indicating that the model developed will be able to give a reasonably good estimate of the response of the system [20].

An analysis of variance (ANOVA) was conducted to check the adequacy and the significance of the quadratic model. According to ANOVA (Table 1), the *F* value determined the significance of each term at the designed level of confidence [21]. The *F* value of percentage of lignin reduction (1166.54) implies that the model is significant. The *p* value was used to check the significance of each variable and simultaneously identify the effect of each factor on the response [22, 23]. According to the *p* values (*p *<* 0.05*), the linear model terms (*A*, *B* and *C*), the interactive model terms (AC), and the quadratic model terms (*C*^2^) were all significant at 95% confidence level. Among the interactions studied, biomass loading and duration resulted in probability value (*p* value) of 0.0198, which was highly significant than other interactions. Furthermore, the variables *A*, *B* and *C* have resulted in significant model terms compared to AB and BC. In this model, only significant terms are considered. The coefficient of variance (CV) specifies the degree of precision for the treatments and expressed as percentage (%). The low values of CV% clearly indicated a very high degree of precision and a good reliability of the experimental values [20]. Percentage of lignin reduction resulted in a lower CV value of 1.92%, which showed that the model has high experimental reliability due to closeness of experimental and predicted values [24].

**Table 1:** Analysis of variance (ANOVA) for quadratic model for lignin reduction of corncob pretreated by HCE-OP_1_

Source	Sum of squares	Degrees of freedom	Mean square	*F* value	*p* value Prob > *F*	Significance
Model	69.87	9	7.76	1166.54	< 0.0001	Significant
*A*	0.99	1	0.99	148.91	< 0.0001	
*B*	0.09	1	0.09	13.6	0.0078	
*C*	65.97	1	65.97	9912.54	< 0.0001	
AB	0.022	1	0.022	3.27	0.1135	
AC	0.06	1	0.06	9.04	0.0198	
BC	1.06E−03	1	1.06E−03	0.16	0.7013	
*A* ^2^	7.52E−07	1	7.52E−07	1.13E−04	0.9918	
*B* ^2^	9.66E−03	1	9.66E−03	1.45	0.2675	
*C* ^2^	2.73	1	2.73	410.21	< 0.0001	
Residual	0.047	7	6.66E−03			
Lack of fit	0.047	3	0.016			
Pure error	0	4	0			
Cor total	69.92	16				
Std. dev.	0.081581			*R*-squared	0.999334	
Mean	4.253575			Adj *R*-squared	0.998477	
C.V. %	1.917932			Pred *R*-squared	0.989339	
Press	0.745405			Adeq precision	103.0419	

Adequate precision measures the signal to noise ratio and compares the range of the predicted values at the design points to the average prediction error. This ratio was greater than 4, which is desirable and indicates adequate model discrimination [25]. In the present case, the adequate precision for a percentage of lignin reduction has resulted in a ratio of 103.04, indicating an adequate signal, implying greater predicted response relative associated error.

**Fig.1:**
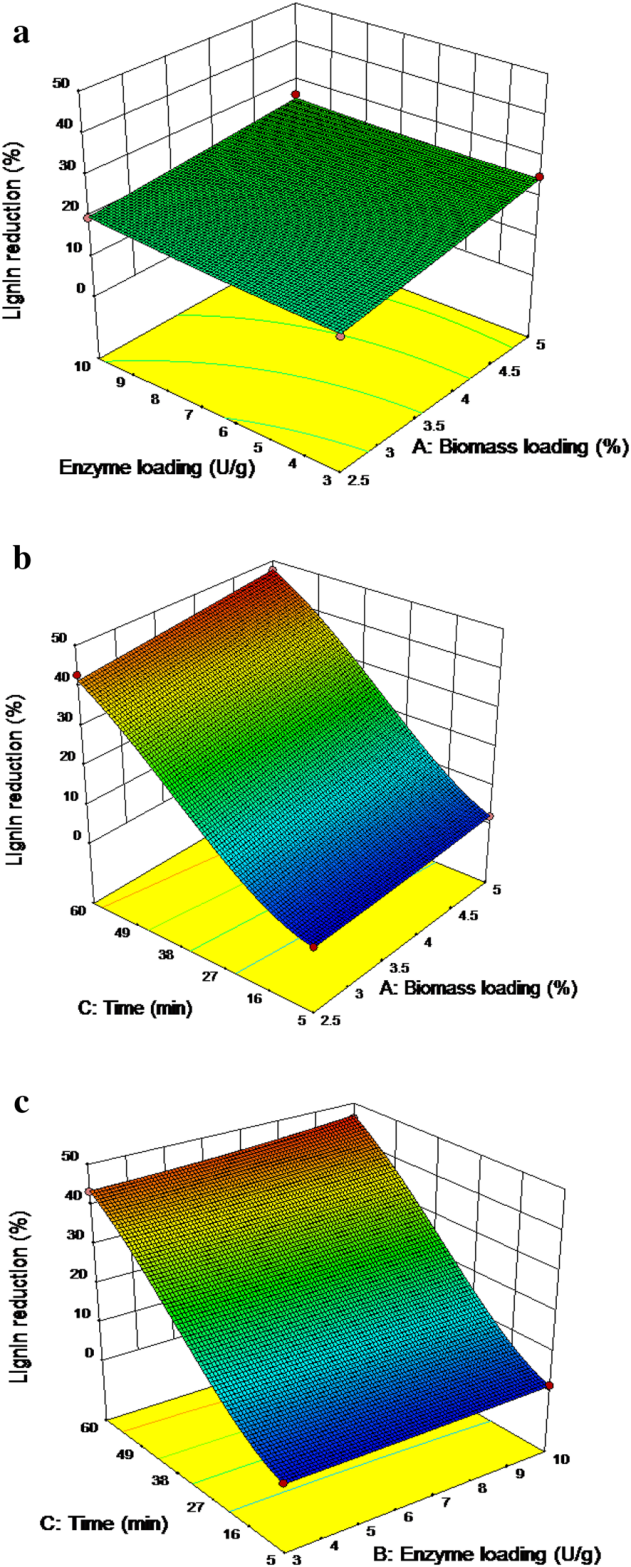
3D plots for response lignin reduction of corncob pretreated by HCE –OP_1_. **a** Enzyme loading vs. biomass loading; **b** biomass loading vs. time; **c** enzyme loading vs. time

The response surface plots were generated with one variable kept at its optimum level and other variables varying within the experimental range. The interactions between the model terms were expressed by a three-dimensional surface. A check for interactions was necessary to determine the significance of the model equation [22, 23]. The response surface plots for a percentage of lignin reduction of the OP_1_ were shown in Fig. 1. An empirical relationship between the response and the variables was expressed by the following quadratic second-order polynomial equation:

$$\begin{aligned} {\text{Lignin reduction}} & = - 1.80307 \, + \, 0.505073 \, \times \, A \, + \, 0.037273 \, \times \, B \, + \, 0.185913 \times \, C \, - \, 0.01686 \\ & \quad \times \, AB \, - \, 0.00357 \, \times \, AC \, + \, 0.000169 \, \times \, BC \, + \, 0.000271 \times \, A^{2} + \, 0.003909 \\ & \quad \times \, B^{2} - \, 0.00106 \, \times \, C^{2} \\ \end{aligned}$$

where *A* is biomass loading in %, *B* is enzyme loading in U g^−1^ of biomass, and *C* is duration in min.

The same experimental designs and conditions were adopted as per OP_1_ for optimizing variables for OP_2_. The optimized conditions obtained in HCE pretreatment with plate 2 (HCE-OP_2_) were the same as plate 1, namely biomass loading (5%), enzyme loading (6.5 U g^−1^) and a process time (60 min). However, in this case, lignin reduction was 35.91%, which was significantly lower than that observed with OP_1_. This might be due to the cavitation bubbles collapse and inward propagation of the shock waves. The geometric focusing of this shock wave at the centre of the cluster creates the enhancement of the cavity cluster collapse (higher pressure) and noise associated with the cavity cluster collapse [26]. This kind of shock wave formation has been confirmed by Wang and Brennen [27] and Reisman et al. [28]. The collapse of micro bubble cavities generates enormous destructive forces, which not only brings about the disintegration of biomass via high turbulence and micro-jets [19, 29], but also due to the dissociation of water molecules, which led to the generation of radicals like HO^−^, HOO^−^, and O_2_^−^. The linear model of lignin reduction (Table 2) showed a predicted and adjusted *R*-squared (*R*^*2*^) values of 0.94 and 0.96, respectively, revealing a good agreement between experimental and predicted values besides implying that the mathematical model was very reliable. The *F* value of 140.32 implies that the model was significant. The compositional analysis of corncob biomass samples after HCE with OP_1_ and OP_2_ are furnished in Additional file 1: Tables S1, S2.

**Table 2:** Analysis of variance (ANOVA) for linear model for lignin reduction of corncob pretreated by HCE –OP_2_

Source	Sum of squares	Degrees of freedom	Mean square	*F* value	*p* value Prob > *F*	Significance
Model	2221.40	3	740.47	140.32	< 0.0001	Significant
*A*	41.32	1	41.32	7.83	0.0151	
*B*	9.95	1	9.95	1.88	0.1930	
*C*	2170.13	1	2170.13	411.25	< 0.0001	
Residual	68.60	13	5.28			
Lack of fit	68.60	9	7.62			
Pure error	0.00	4	0			
Cor total	2290.00	16				
Std. dev.	2.30		*R*-squared	0.9700		
Mean	17.11		Adj *R*-squared	0.9631		
C.V. %	13.43		Pred *R*-squared	0.9430		
Press	130.45		Adeq precision	33.6410		

**Fig.2:**
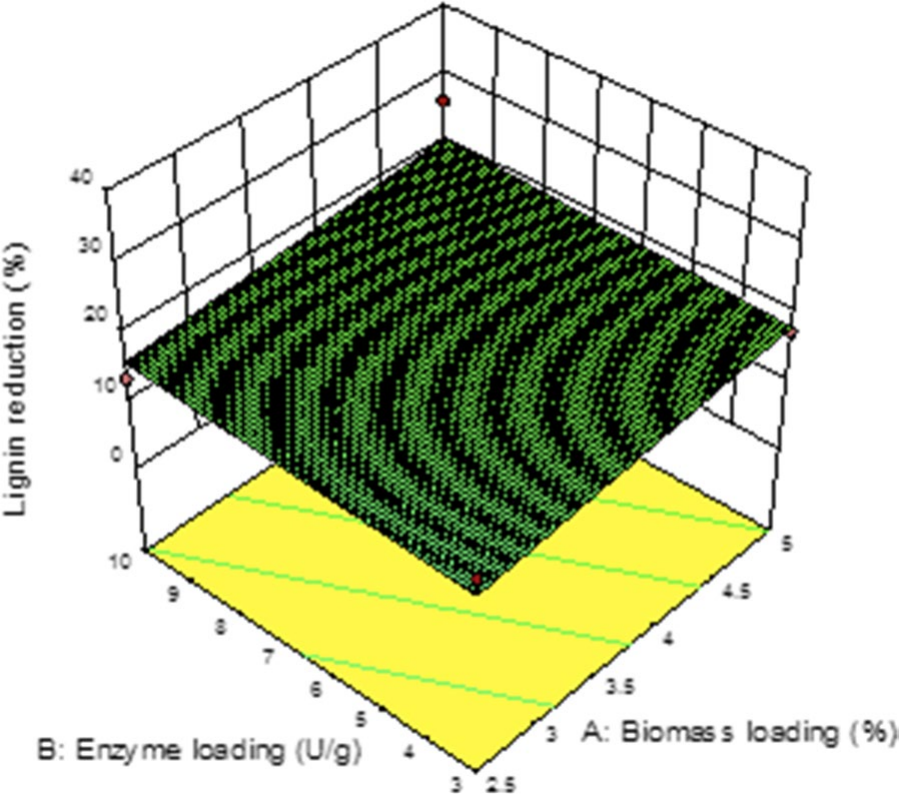
3D plots for response lignin reduction of corncob pretreated by HCE-OP_2_—enzyme loading vs. biomass loading

According to the *p* values (*p* < 0.05), the linear model terms (*A* and *C*) are significant at 95% confidence level. The CV value of 13.43 indicated that the model had reliability of the experimental values. An adequate precision ratio of 130.45 represents an adequate signal. The response surface plots are shown in Fig. 2. The empirical equation fitting the quadratic model is given below:

$$\text{Lignin reduction} = \text{1}{.818182 \times A } + 0{.318588 \times B} + 0{.598915 \times C}$$

where *A* is biomass loading in %, *B* is enzyme loading in U g^−1^ of biomass, and *C* is duration in min.

The results of the present study are compared with different HC biomass pretreatment methods which is shown in the Table 3. The present experiment achieved 47% lignin recovery with a lower optimal temperature of operation. More importantly, the energy consumption for the HCE pretreatment process is very low compared to other reported methods.

**Table 3:** Comparison of different HC biomass pretreatment methods

S. no.	Parameters	Hilares et al. [30]	Hilares et al. [18]	Kim et al. [19]	Present study
1	Biomass	Sugarcane bagasse	Sugarcane bagasse	Reed	Corncob
2	Feedstock size	4.7 mm	1.18 - 1.70 mm	10 mm	≤ 212 µm
3	Type of plate	Orifice	Orifice	Orifice	Orifice
4	Number of holes	16 ($\emptyset$ = 1 mm)	27 ($\emptyset$ = 1 mm)	27 ($\emptyset$ = 1 mm)	9 ($\emptyset$ = 2 mm)
5	Operating temperature, °C	70	64	77	30
6	Inlet pressure, kPa	300	300	500	50
7	Reaction time, min.	30	44.48	41.1	60
8	Biomass loading, %	–	4.27	11.8	5.0
9	Catalyst	0.3 M NaOH	0.48 M NaOH	3.0% NaOH	Laccase enzyme: 6.5 U g^−1^ of biomass
10	Liquid volume, ml	2500	2500	150	4000
11	Biomass placement	Cavitation zone (Cylindrical wire cloth: 18 mesh)	Cavitation zone (Cylindrical wire cloth: 18 mesh)	Cavitation zone (woven wire cloth: 40 mesh)	Mixed with acetate buffer and circulated in a closed loop
12	Lignin removal, %	51.52	60.4	35–42	47.44
13	Cavitational yield, g J^−1^	–	–	–	3.56 × 10^−5^
15	Energy consumption, MJ kg^−1^	–	–	3.65	1.35

### Thermal behavior of raw and pretreated biomass

**Fig. 3:**
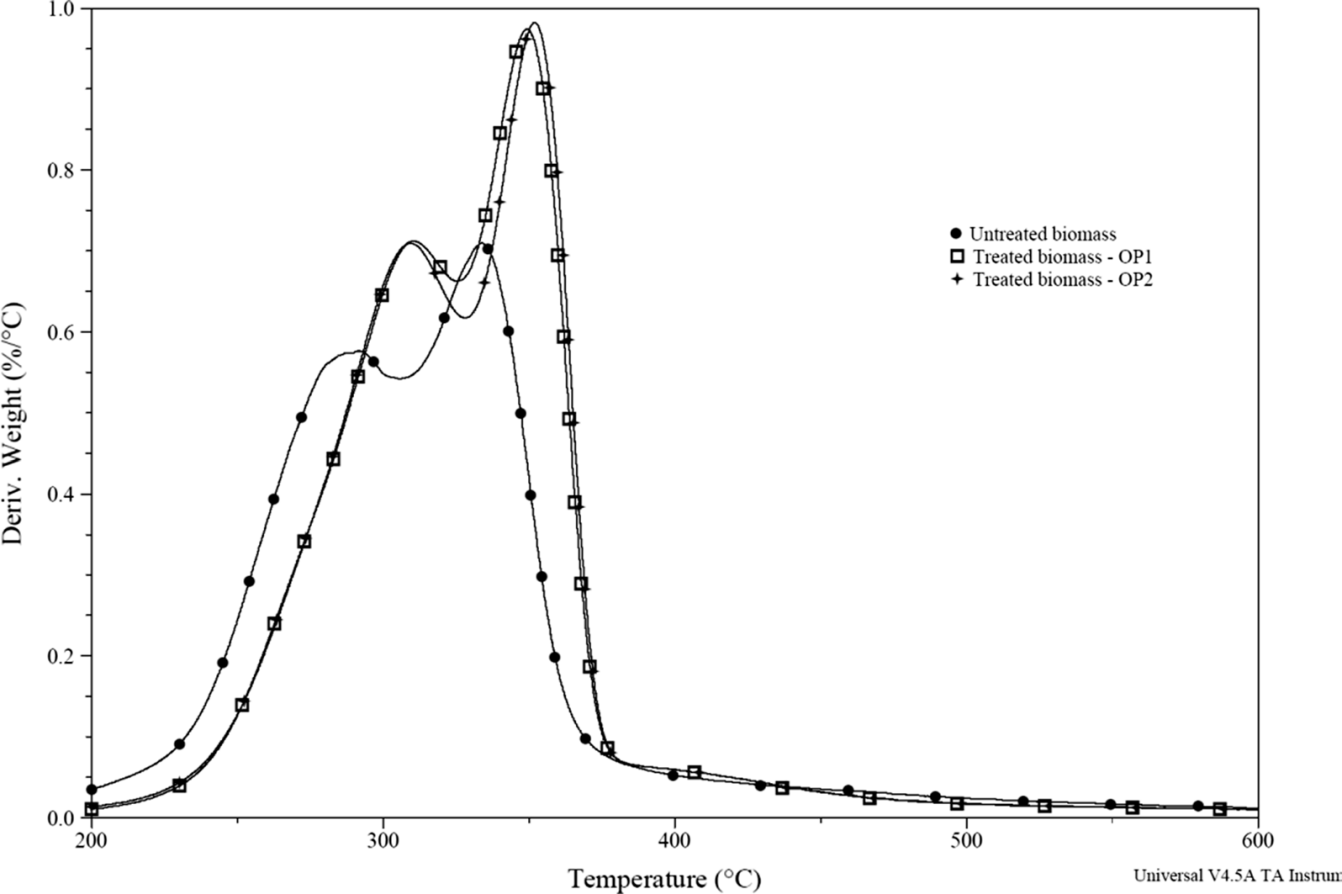
Combined DTG curves for raw and HCE pretreated corncob biomass samples

Thermogravimetric analysis of lignocellulosic biomass was used to study the thermal degradation profiles of hemicellulose (250–300 °C), cellulose (300–350 °C), and lignin (300–500 °C) [31, 32]. The appearance of the derivative thermogravimetry (DTG) peak at 200–400 °C was pertaining to hemicellulose and cellulose component [31]. This information enables comparison of the changes in composition of biomass due to pretreatment method [33, 34]. Furthermore, it was clear that the two peaks observed between 200 and 400 °C in the DTG curves was associated with the degradation of hemicellulose and cellulose of the sample. Perhaps the ranges of temperature for decomposition of lignin would have overlapped partially with that of hemicelluloses and cellulose. It is obvious that the temperature associated with two DTG peaks of pretreated corncob samples was higher than raw sample and this could be due to pretreatment effect (Fig. 3 and Table 4).

**Table 4:** Details of temperature at peak 1 and peak 2 for corncob biomass obtained from DTG curves

Type of biomass	Catalyst used	Temperature, °C (maximum loss rate, %/ °C)	
		Peak 1	Peak 2
Raw corncob	–	290.7 (0.5753%/ °C)	333.7 (0.7088%/ °C)
Pretreated biomass (HCE-OP_1_)	6.5 U of enzyme g^−1^ of dry biomass	310.9 (0.7122%/ °C)	349.0 (0.9737%/ °C)
Pretreated biomass (HCE-OP_2_)	6.5 U of enzyme g^−1^of dry biomass	310.4 (0.7092%/ °C)	352.0 (0.9820%/ °C)

Apart from temperature change, the peaks also differed in position and height indicating changes in proximate composition namely reduction in lignin and hemicellulose and increase in cellulose content, that might be happened during pretreatment process.

### FT-IR analysis

Pretreated sample was analyzed by Fourier-transform infrared (FT-IR), and the different wave numbers, functional groups and their corresponding polymer were presented in Table 5. It is evident that raw corncob showed clear peaks for cellulose, hemicellulose and lignin at the corresponding wave numbers.

**Table 5:** Assignment of functional groups and their corresponding polymers in pretreated corncob

Wavenumber	Functional groups	Corresponding polymer
3340	O–H stretch	Lignin
2833	C–H stretch	Lignin
1634	Aromatic ring vibration	Lignin
1509	C=C	Lignin
1422	CH_2_	Lignin
1321	C–O–CH vibration	Lignin
1247	C–O stretching	Syringyl units
1157	C–O–C asymmetrical stretching	Hemicellulose (xylose)
1031	C–O,C=C,C–C–O stretching	Cellulose, hemicellulose and lignin

The performance of combined HCE pretreatment indicated that there were no major changes in hemicellulose. However, both the orifice plates behaved differently with regard to lignin removal.

**Fig. 4:**
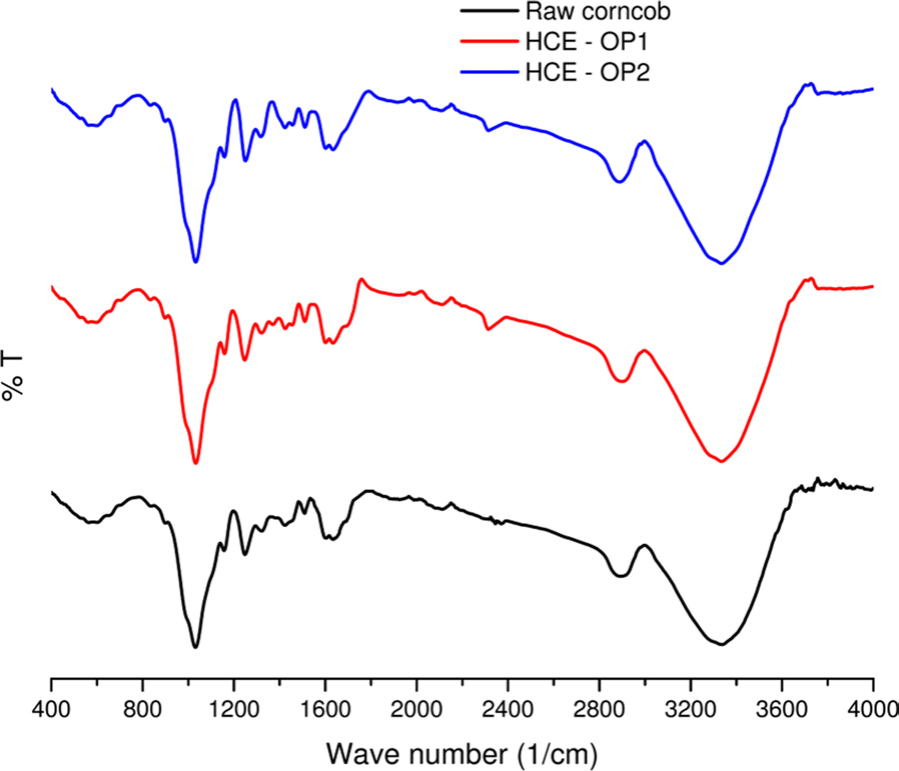
FTIR spectrum for raw and HCE pretreated corncob samples

A reduction in peak intensity was observed under OP_1_ than OP_2_ compared to the raw biomass, at wave numbers 1320, 1424, 1445, and 1509 cm^−1^ (Fig. 4). This signifies that lignin removal was higher in OP_1_, and OP_2_ was less efficient. Probably this difference between the plates could be attributed to the less number of radicals formed in the cavitational zone by the OP_2_. Moreover, other functional groups also showed similar changes in both OP_1_ and OP_2_.

### Scanning electron microscopy (SEM)

**Fig. 5:**
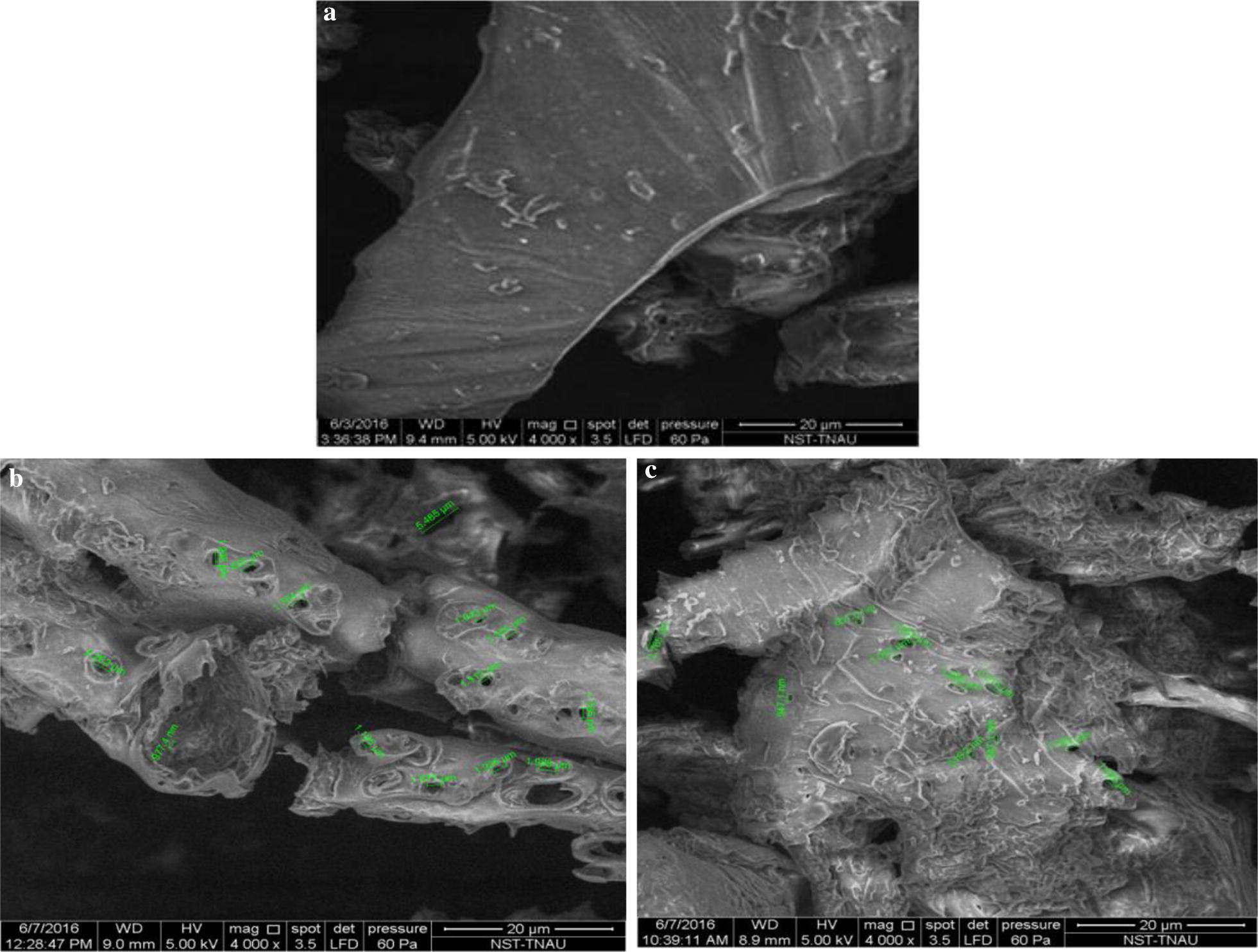
SEM images **a** untreated corncob, **b** corncob treated by HCE-OP_1_, **c** corncob treated by HCE-OP_2_

The SEM images of raw sample shows a smooth and undisturbed surface (Fig. 5a). By contrast, after the HCE pretreatment, pores appeared on the surface, and it could be due to removal of lignin. According to Grimaldi et al. [35], the biomass pretreatment removes lignin through destruction of cell wall in two stages namely, loss of cohesion between neighboring cell walls, and degradation inside the cell wall by peeling off and forming holes. The breakdown of structure and pinholes in the surface area of treated sample by cavitation could have enhanced the accessibility for hydrolytic enzymes for increasing the saccharification process [36]. HCE-generated potholes in biomass might have occurred due to collapse of the cavities at the surface of the biomass particles, which could lead to a shear of bimolecular structures, while subsequent laccase treatment led to increase in pothole size (1–6 µm). This indicates effective removal of lignin and deconstruction of biomass in OP_1_ than OP_2_ (max. 2 µm) (Fig. 5b, c), which could be attributed to the higher intensity of cavities collapse and turbulence generated in OP_1_ that resulted in a loosening of the biomass for the effective action of laccase than in plate 2.

### X-ray diffraction (XRD)

Raw and pretreated samples were estimated for cellulose crystallinity changes. The value of crystallinity index of pretreated samples (56.0%) for both the orifice plates were higher than the raw sample (52.4%), indicating that HCE pretreatment was more efficient in the removal of amorphous fraction as compared to crystalline fractions. No significant change in the crystallinity of cellulose between treatments and type of plates was observed. This observation testifies that laccase removed only lignin and not changed the cellulose crystallinity. Instead, the HCE pretreatment improved the accessibility of cellulose without modifying the cellulose structure.

### Cavitational yield and energy consumption

The optimized conditions for biomass treatment in HCE pretreatment with OP_1_ and OP_2_ were biomass loading at 5%, enzyme loading at 6.5 U g^−1^of biomass, and a process time of 60 min. Energy consumption in the HCE pretreatment process for OP_1_ (1.35 MJ kg^−1^) was lower than OP_2_ (3.24 MJ kg^−1^) with cavitational yields of 3.56 × 10^−5^ and 2.70 × 10^−5^ g J^−1^, respectively, under optimized conditions for OP_1_ and OP_2_ for the enzyme pretreatment.

## Materials

### Biomass preparation

The corncob biomass was procured from the farmer fields, Coimbatore, Tamil Nadu, India and dried at ambient temperature (30 °C) for 12 h to reduce the moisture content from 20 to 14%, then the size was reduced in a sequence through shredder (Chudekar Agro Engg Pvt Ltd., Model: 53 H, India), pin mill (Premium Pulman Pvt Ltd., Model: PPM-12, India) and grinder (Aashapura Enterprises, Model: Stylo 750, India) to ≤ 212 µm, which was sieved via ASTM sieve size No. 70.

### Enzyme selection

Generally, two families of ligninolytic enzymes are widely considered for enzymatic delignification, which includes phenol oxidase (laccase) and peroxidases (lignin peroxidase, or LiP and manganese peroxidase, or MnP) [37]. Laccase belongs to the copper oxidase enzyme family, similar to other phenol-oxidizing enzymes and it preferably polymerizes lignin by coupling of the phenoxy radicals produced from oxidation of lignin phenolic groups. Laccase enzyme was selected and used as catalyst for this HCE pretreatment [38].

The enzyme laccase used in the study was from microbial source (Trametes versicolor). The laccase was purchased from Sigma-Aldrich, Bangalore, India and used as such. The laccase activity was determined with the reaction mixture contained appropriately diluted enzyme mixed with 1 mM ABTS in sodium phosphate buffer (50 mM, pH 4.5) at 30 °C for 5 min. using 1 mM ABTS by monitoring changes in absorbance at 420 nm (€ max = 3.6 × 104 M^−1^ cm^−1^) spectrophotometrically in a Spectramax 360 (Molecular devices, USA). One unit of enzyme activity refers to the amount of enzyme required to oxidize 1 µM min^−1^ of the ABTS substrate under standard assay conditions.

### System description and operating conditions

**Fig. 6:**
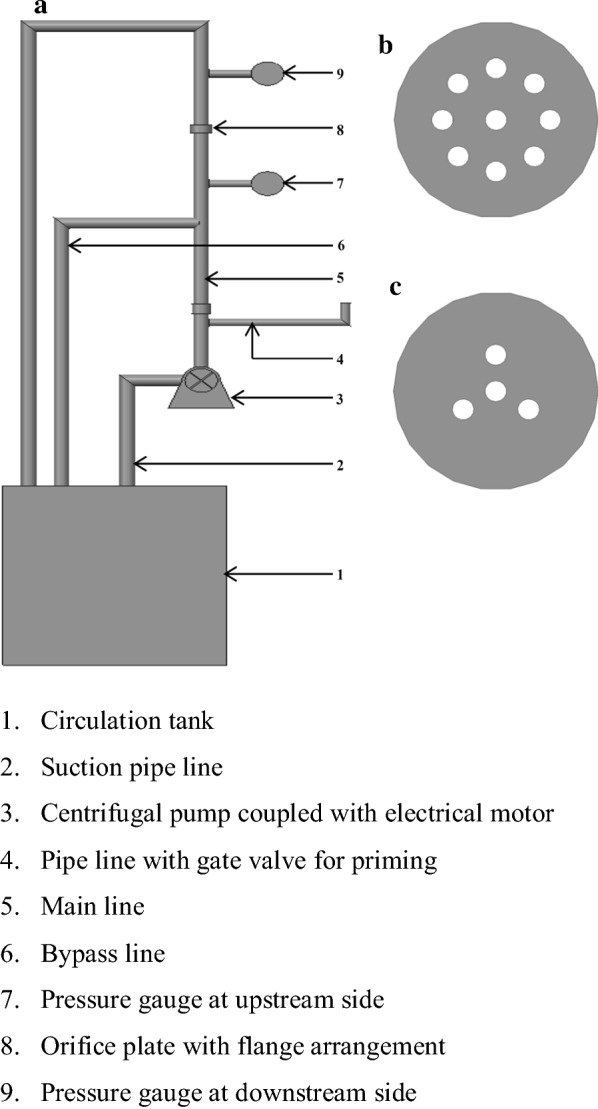
**a** Hydrodynamic cavitation reactor, **b** orifice plate 1, **c** orifice plate 2

The main hurdles associated with commercialization of higher biomass loading pretreatment reactors are complexity of design, reactor geometry and upscale, poor mixing characteristics of reactants, and it is an energy-intensive process. For these reasons, low biomass loading rate (< 20%) is largely preferred in most of the biomass pretreatments [39]. The main criteria to be considered for reactor design is rheological properties of different slurries collected from different unit operations in the fermentation process [40]. Mostly, water with catalyst is used as the working fluid in the recent HC biomass pretreatment studies; whereas, in the present study corncob biomass slurry (buffer + biocatalyst + powdered biomass) was used as the working liquid. The process design of this pretreatment is to supply the biomass slurry via holes in the orifice plate to cavitation zone. The rheological study involving different corncob biomass slurries (2.50, 3.75, 5.00, 6.75, 7.50, 8.75 and 10.00%) showed increased viscosity and yield stress, along with increased biomass solid loading and these slurries exhibit pseudo-plastic or shear-thinning behavior [41]. It is also observed that high biomass loading rate (> 6.75%) of corncob slurry have blocked the holes in orifice plate. Based on this result, low biomass loading rates (2.50, 3.75 and 5.00%) were selected for HCE pretreatment. Hydrodynamic cavitation reactor (HCR) consists of circulation tank (6 L capacity), orifice plate, flanges for orifice plate, centrifugal pump, electrical motor, gate valves for priming and bypass, pressure gauges, and pipe accessories (Fig. 6a). Two pressure gauges (*P*_1_ and *P*_2_) were fixed on both the downstream and upstream sides of the orifice plate to measure the pressure drop. For same area opening in the orifice plate, higher diameter of the hole is recommended for intensive cavitation applications and vice versa. In this reactor, total hole area openings made in the plate were kept as constant (28.26 mm^2^) and for constant area openings, two configurations of orifice plates were used, viz., orifice plate 1 (OP_1_:9 holes and 2 mm Ø) and orifice plate 2 (OP_2_:4 holes and 3 mm Ø) (Fig. 6c). The suction pipe of the pump was connected at the base of the circulation tank. The main pipeline was divided into three sub-pipelines to serve for three purposes namely priming, bypass line, sub mainline to accommodate flanges and orifice plate, and it was connected to the delivery pipe of the centrifugal pump. To make a closed loop circulation, the working fluid was supplied from the circulation tank to the orifice plate with the help of a centrifugal pump and then sent back to circulation tank. The purpose of the bypass valve was to regulate the flow rate/pressure of the working fluid (biomass slurry) by passing through the orifice plate of the reactor.

For HCE biomass pretreatment, the biomass slurry was prepared by mixing appropriate quantities of biomass powder and a biocatalyst (laccase enzyme) in an acetate buffer pH 4.5. For example, 200 g of biomass and 6.5 U g^−1^ of enzyme were added to 4000 mL of acetate buffer to make biomass slurry for 5% biomass loading and 6.5 U g^−1^ of enzyme. The prepared slurry was added to circulation tank and made to circulate via selected orifice plate continuously, which enabled the biomass to get exposed to cavitation action. Cavitation involves production and aggressive collapse of micro bubbles to generate more hotspots, having higher temperature and pressure. This is sufficient to make the chemical and physical transformations in the lignocellulosic biomass. During the process, decomposition of water molecule causes free radical formation, such as hydroxyl radicals leading to turbulence action of working fluid in the cavitation zone, which eventually helps in rupturing the lignin barrier. Laccase enzyme can oxidize a variety of phenolic subunits of lignin and other aromatic compounds via radical-catalyzed mechanism to yield oxygen-centred free radical and quinine for subsequent reduction in the polymerization reaction [42–44]. Hence, HCE-based biomass treatment helps in the generation of highly reactive free radicals in HCR, which can improve the mass transport process rates and enhance the lignin degradation. Based on these results, three biomass loadings (2.50, 3.75 and 5.00%) were selected for HCE pretreatment.

### Compositional analysis

After each run, the biomass slurry was collected from the circulation tank, and filtered via filter cloth to separate the pretreated biomass from supernatant. The supernatant was stored at 4 °C for further analysis. The pretreated biomass was washed twice with distilled water to obtain a neutral pH of 7.0 and the biomass samples were dried in a hot air oven at 45 °C for compositional analysis. National Renewable Energy Laboratory procedure was adopted for analyzing biomass composition of raw and pretreated samples [45]. The percentage of lignin reduction by the pretreatment process was calculated using the following equation.

$$\text{Percentage of lignin reduction = }\frac{{\text{lignin in raw biomass{-}lignin in pretreated biomass}}}{{\text{lignin in the raw biomass}}} \times 100$$

### Experimental design

Response surface methodology (RSM) is a statistical and mathematical technique widely used for optimization of process parameters and their interactions on output response(s). The added advantages of optimization via RSM approach are (i) only a minimum number of trials are sufficient to find optimum, (ii) to find a correlation between independent inputs and output responses and (iii) reduction in time, materials and cost because of the less number of trials are needed [46]. The optimization of biomass pretreatment process involves studying the influence of operational parameters and their interactions on lignin removal from raw biomass. For enzymatic biomass pretreatment, process parameters such as biomass loading, enzyme loading and reaction time are crucial factors in deconstruction of the lignin structure as well as lead to release of reducing sugars due to solubilization of biomass.

Response surface methodology (RSM) was employed to determine the optimal conditions for HCE pretreatment to attain the maximum percentage of lignin reduction in the corncob biomass samples. The response was assumed to be influenced by three independent variables, catalyst concentration (*A*), biomass to liquid ratio (*B*) and reaction time (*C*). The range of three selected independent variables used for pretreatment process are: biomass loading of 2.5–5.0%, enzyme loading of 3–10 U g^−1^ of dried biomass, and reaction time of 5–60 min. Based on the results of the preliminary trials, the above range of levels of the three independent variables were fixed. A total of 17 experimental trials of the three variables were designed by Box–Behnken design via Design-Expert software 10.0 (Stat-Ease, Inc., USA) [47].

### Structural composition of biomass

#### FT-IR analysis

The FT-IR spectra of the tested biomass samples were obtained using an FT-IR (FT-IR 6800 JASCO, Japan). Absorbance spectra were recorded between 4000 and 400 cm^−1^ wave numbers with a spectral resolution of 4 cm^−1^ and 64 scans per sample.

#### SEM analysis

The morphology of raw and pretreated corncob biomass was analyzed by scanning electron microscope (SEM; Quanta 250, FEI, Hillsboro, OR, USA) using an Everhart–Thornley Detector (ETD) detector. The SEM was operated in a vacuum, 10 kV, with a spot size of 4 and a pressure of 20 Pa. The sample images were taken at ×4000 magnification.

#### XRD analysis

The cellulose crystallinity of the biomass samples was measured using an Ultima IV diffractometer (Rigaku, Japan). Copper K*α* radiation, 30.0 kV of voltage, 15 mA of current, and a rate of 2.0°min^−1^ for a 2*θ* continuous scan from 4.0° to 70.0° were applied. The crystallinity index was obtained from the ratio of the maximum peak intensity 002 (*I*_002_, 2*θ* = 22.0) and minimal depression (*I*_am_ 2*θ* = 16.5) between peaks 001 and 002 [48, 49].

$${\text{Crystallinity index}} = \frac{{I_{002} - I_{\text{am}} }}{{I_{002} }} \times 100$$

where *I*_002_ is the diffraction intensity at 2*θ* = 22.5°, which represents both the crystalline and amorphous regions, and *I*_am_ is the diffraction intensity at 2*θ* = 18.5°, which represents the amorphous regions.

#### Thermogravimetric analysis (TGA)

The sample size of the corncob used in the experiment was 10 ± 2 mg in a thermogravimetric analyzer (TA instruments, Model: TGA Q50, USA). The test sample was heated at a heating rate of 10 °C min^−1^ for the temperature range from 50 to 800 °C and nitrogen gas was purged at a flow rate of 30 mL min^−1^ to create pyrolysis conditions. TGA curve was plotted using TA software (TA Universal Analysis 2000) for both raw and pretreated samples and the results were compared.

### Effect of cavitation on temperature of working liquid and residual enzyme activity

During the experimental trials, the temperature of the working liquid was measured at an interval of 5 min by digital thermometer (Multi-thermometer, India). Similarly, the residual enzyme activity during the reaction period was also determined [50].

#### Working liquid temperature

The acetate buffer was used as working liquid in the HCE biomass pretreatment and there was a rise in the temperature of the working liquid from 30 to 50 °C in 60 min, which could be attributed to heat energy dissipation by sudden collapses of bubbles and cavities. Since laccase enzyme was used, the temperature of working liquid was maintained at 30 °C by circulating cold water to circulation tank during the experiment.

#### Residual enzyme activity

Enzyme’s protein conformation changes in temperature, pH, ion concentration, and mechanical stress, and microenvironment of a solution. In this study it was observed that, the laccase enzyme was still active (OP_1_:23.5%, OP_2_:32.05%) after HCE treatment, this implies that the residual enzyme can be reused for another batch of pretreatment. For OP_1_, operated with inlet pressure of 50 kPa, the residual enzyme activities at 5, 10, 20 and 30 min were 34.2, 28.6, 23.5, and 23.5%, respectively. The residual enzyme activity was initially reduced and stabilized after 30 min. While OP_2_ operating with inlet pressure of 100 kPa showed that the residual enzyme activity was gradually reduced over time (34.2, 33.1, 32.1 and 32.05% in 5, 10, 20 and 30 min, respectively). Typically, the residual enzyme activity obtained for OP_1_ was less than that of OP_2_, possibly due to variation in higher inlet pressure, coupled with hole numbers and diameter. The frequency of turbulence decreases with an increase in the diameter of the orifice hole [51]. Laccase, being a green catalyst, is widely used for delignification. Although it contributes for the cost factor, in this study, we have shown that laccase after HCE treatment did not lose its activity much. This indicates that the enzyme can be reused for further pretreatment.

### Cavitational yield

The cavitational yield is the result of several design parameters optimized for cavitating reactor [52, 53]. This yield can be enhanced by changing flow conditions and reactor geometry. The orifice plates with higher holes results higher cavitational yield due to increased cavitational effects. Cavitational yield can be defined as number of molecules degraded per unit energy dissipated.

$${\text{Cavitational yield}}\, = \,8.834\, \times \,10^{ - 11} \left( {P_{\text{collapse}} } \right)^{1.1633}$$

Collapse pressure can be predicted by

$$P_{\text{collapse}} \, = \,7527 \, \left( F \right)^{ - 2.55} \, \times \,\left( {P_{I} } \right)^{2.46} \left( {R_{0} } \right)^{ - 0.80} \left( {d_{o} } \right)^{2.37}$$

where *R*_0_ is the initial cavity size, mm; *P*_I_ is the inlet pressure, atm; Do is the diameter of the hole in the orifice plate, mm; *F* is the percentage free area of holes in the total cross-sectional area of the pipe

### The energy required per kg of biomass in an HCE pretreatment process

The energy consumed for treating one kg of biomass by the HCE pretreatment process was calculated by the following equation.

$${\text{Energy consumption, MJ/kg}} = \frac{{{\text{Inlet pressure}} \left( {\frac{N}{{m^{2} }}} \right) \times \;{\text{flowrate }}\left( {m^{3} } \right) \times {\text{reaction time}}\left( s \right)}}{{{\text{Weight of biomass used }}\left( {\text{kg}} \right)}}$$

## Conclusion

Based on the results it is clear that HCE pretreatment process have decreased the lignin content and increased the cellulose recovery from corncob. The optimum conditions were 5% of biomass loading, 6.5 U g^−1^ of enzyme loading and the process time of 60 min to get a maximum reduction in lignin content. The percent reduction of lignin by OP_1_ and OP_2_ were 47.44 and 35.91%, respectively. This study provides evidence that HCE pretreatment is more efficient in decreasing the lignin content. However, for scaling up, 5% of biomass loading seems to be low and may be increased to achieve an economic output. We hypothesize that it can be accomplished by altering the orifice plate configuration and operating pressure. The conformity studies (TGA, FT-IR, SEM and XRD) have confirmed the deconstruction of biomass, removal of lignin and increase in cellulose accessability by HCE pretreatment. Overall, HCE pretreatment technology is advantageous than other pretreatment method because it requires lesser energy and easy to scale up, and can be used in conjunction with generation of biofuel and other LC-derived bioproducts from biomass as a pretreatment process.

## Additional file

**Additional file 1: Table S1.** Results of compositional analysis of pretreated biomass and lignin reduction (%) for HCE with OP_1_. **Table S2.** Results of compositional analysis of pretreated biomass and lignin reduction (%) for HCE with OP_2._

## Abbreviations

ANOVA: analysis of variance
CrI: crystallinity index
CV: coefficient of variance
DTG: derivative thermogravimetry
FT-IR: Fourier-transform infrared
HC: hydrodynamic cavitation
HCE: hydrodynamic cavitation and enzymatic pretreatment
HCR: hydrodynamic cavitation reactor
LC: lignocellulosics
LCB: lignocellulosic biomass
NREL: National Renewable Energy Laboratory
OP_1_: orifice plate 1
OP_2_: orifice plate 2
RSM: response surface methodology
SEM: scanning electron microscopy
TGA: thermogravimetric analysis
XRD: X-ray diffraction
