# Supplementary material for: Delignification of corncob via combined hydrodynamic cavitation and enzymatic pretreatment: process optimization by response surface methodology
Source: Biotechnol Biofuels. 2018 Jul 24;11:203. doi: 10.1186/s13068-018-1204-y (PMC6057035; doi:10.1186/s13068-018-1204-y)
Supplement: Supplementary file 1 — Additional file 1: Table S1. Results of compositional analysis of pretreated biomass and lignin reduction (%) for HCE with OP1. Table S2. Results of compositional analysis of pretreated biomass and lignin reduction (%) for HCE with OP2. [file 13068_2018_1204_MOESM1_ESM.docx]

**Additional file 1 Additional supporting data.**

**Table S1 Results of compositional analysis of pretreated biomass and lignin reduction (%) for HCE with OP_1_**

| Std | Run | Biomass loading (%) | Enzyme loading (U/g of biomass) | Time (min) | Lignin (%) | Hemicellulose (%) | Cellulose (%) | Lignin reduction (%) |
| --- | --- | --- | --- | --- | --- | --- | --- | --- |
| 13 | 1 | 3.75 | 6.5 | 32.5 | 13.86 | 27.12 | 41.05 | 21.25 |
| 5 | 2 | 2.50 | 6.5 | 5.0 | 17.56 | 27.30 | 36.10 | 0.23 |
| 1 | 3 | 2.50 | 3.0 | 32.5 | 14.72 | 27.28 | 40.58 | 16.36 |
| 9 | 4 | 3.75 | 3.0 | 5.0 | 17.44 | 27.32 | 36.27 | 0.91 |
| 12 | 5 | 3.75 | 10.0 | 60.0 | 9.50 | 26.68 | 44.95 | 46.02 |
| 8 | 6 | 5.00 | 6.5 | 60.0 | 9.25 | 26.50 | 45.14 | 47.44 |
| 3 | 7 | 2.50 | 10.0 | 32.5 | 14.12 | 26.97 | 40.78 | 19.77 |
| 11 | 8 | 3.75 | 3.0 | 60 | 9.98 | 27.01 | 43.78 | 43.30 |
| 15 | 9 | 3.75 | 6.5 | 32.5 | 13.86 | 27.12 | 41.05 | 21.25 |
| 10 | 10 | 3.75 | 10.0 | 5.0 | 17.39 | 27.05 | 36.52 | 1.19 |
| 7 | 11 | 2.50 | 6.5 | 60.0 | 10.05 | 27.26 | 43.54 | 42.90 |
| 4 | 12 | 5.00 | 10.0 | 32.5 | 12.98 | 26.96 | 41.82 | 26.25 |
| 17 | 13 | 3.75 | 6.5 | 32.5 | 13.86 | 27.12 | 41.05 | 21.25 |
| 2 | 14 | 5.00 | 3.0 | 32.5 | 13.17 | 27.16 | 41.25 | 25.17 |
| 6 | 15 | 5.00 | 6.5 | 5.0 | 17.3 | 27.05 | 36.48 | 1.70 |
| 14 | 16 | 3.75 | 6.5 | 32.5 | 13.86 | 27.12 | 41.05 | 21.25 |
| 16 | 17 | 3.75 | 6.5 | 32.5 | 13.86 | 27.12 | 41.05 | 21.25 |

**Table S2. Results of compositional analysis of pretreated biomass and lignin reduction (%) for HCE with OP_2_**

| Std | Run | Biomass loading (%) | Enzyme loading (U/g of biomass) | Time (min) | Lignin (%) | Hemicellulose (%) | Cellulose (%) | Lignin reduction (%) |
| --- | --- | --- | --- | --- | --- | --- | --- | --- |
| 9 | 1 | 2.50 | 6.5 | 5.0 | 17.45 | 27.24 | 36.45 | 0.85 |
| 10 | 2 | 5.00 | 6.5 | 5.0 | 17.35 | 27.25 | 36.58 | 1.42 |
| 14 | 3 | 3.75 | 6.5 | 32.5 | 14.88 | 26.65 | 40.08 | 15.45 |
| 6 | 4 | 3.75 | 10.0 | 5.0 | 17.38 | 27.04 | 36.88 | 1.25 |
| 13 | 5 | 3.75 | 6.5 | 32.5 | 14.88 | 26.65 | 40.08 | 15.45 |
| 8 | 6 | 3.75 | 10.0 | 60.0 | 11.42 | 26.08 | 43.60 | 35.11 |
| 17 | 7 | 3.75 | 6.5 | 32.5 | 14.88 | 26.65 | 40.08 | 15.45 |
| 7 | 8 | 3.75 | 3.0 | 60.0 | 11.86 | 26.45 | 42.92 | 32.61 |
| 4 | 9 | 5.00 | 10.0 | 32.5 | 13.01 | 26.12 | 40.12 | 26.08 |
| 15 | 10 | 3.75 | 6.5 | 32.5 | 14.88 | 26.65 | 40.08 | 15.45 |
| 11 | 11 | 2.50 | 6.5 | 60.0 | 11.89 | 26.75 | 42.78 | 32.44 |
| 3 | 12 | 5.00 | 3.0 | 32.5 | 14.51 | 26.72 | 39.87 | 17.56 |
| 12 | 13 | 5.00 | 6.5 | 60.0 | 11.28 | 26.08 | 43.82 | 35.91 |
| 16 | 14 | 3.75 | 6.5 | 32.5 | 14.88 | 26.65 | 40.08 | 15.45 |
| 5 | 15 | 3.75 | 3.0 | 5.0 | 17.46 | 27.32 | 36.28 | 0.80 |
| 1 | 16 | 2.50 | 3.0 | 32.5 | 14.78 | 27.04 | 39.67 | 16.02 |
| 2 | 17 | 2.50 | 10.0 | 32.5 | 15.23 | 26.76 | 40.07 | 13.47 |
